# Inhibition of γ-secretase worsens memory deficits in a genetically congruous mouse model of Danish dementia

**DOI:** 10.1186/1750-1326-7-19

**Published:** 2012-04-26

**Authors:** Robert Tamayev, Luciano D’Adamio

**Affiliations:** 1 Department of Microbiology & Immunology, Albert Einstein College of Medicine, Bronx, NY, 10461, USA

## Abstract

**Background:**

A mutation in the *BRI2/ITM2b* gene causes familial Danish dementia (FDD). BRI2 is an inhibitor of amyloid-β precursor protein (APP) processing, which is genetically linked to Alzheimer’s disease (AD) pathogenesis. The FDD mutation leads to a loss of BRI2 protein and to increased APP processing. APP haplodeficiency and inhibition of APP cleavage by β-secretase rescue synaptic/memory deficits of a genetically congruous mouse model of FDD (FDD_KI_). β-cleavage of APP yields the β-carboxyl-terminal (β-CTF) and the amino-terminal-soluble APPβ (sAPPβ) fragments. γ-secretase processing of β-CTF generates Aβ, which is considered the main cause of AD. However, inhibiting Aβ production did not rescue the deficits of FDD_KI_ mice, suggesting that sAPPβ/β-CTF, and not Aβ, are the toxic species causing memory loss.

**Results:**

Here, we have further analyzed the effect of γ-secretase inhibition. We show that treatment with a γ-secretase inhibitor (GSI) results in a worsening of the memory deficits of FDD_KI_ mice. This deleterious effect on memory correlates with increased levels of the β/α-CTFs APP fragments in synaptic fractions isolated from hippocampi of FDD_KI_ mice, which is consistent with inhibition of γ-secretase activity.

**Conclusion:**

This harmful effect of the GSI is in sharp contrast with a pathogenic role for Aβ, and suggests that the worsening of memory deficits may be due to accumulation of synaptic-toxic β/α-CTFs caused by GSI treatment. However, γ-secretase cleaves more than 40 proteins; thus, the noxious effect of GSI on memory may be dependent on inhibition of cleavage of one or more of these other γ-secretase substrates. These two possibilities do not need to be mutually exclusive. Our results are consistent with the outcome of a clinical trial with the GSI Semagacestat, which caused a worsening of cognition, and advise against targeting γ-secretase in the therapy of AD. Overall, the data also indicate that FDD_KI_ is a valuable mouse model to study AD pathogenesis and predict the clinical outcome of therapeutic agents for AD.

## Background

AD is characterized by amyloid deposition of Aβ peptides that derive from sequential cleavage of APP by β- and γ-secretases [1,2]. Mutations in *APP* cause familial AD (FAD) [3]. Familial dementia is also caused by mutations in genes that regulate APP processing. These include the *PSEN1/2* genes, which code for the catalytic component of the γ-secretase, and the *BRI2/ITM2b* gene, whose protein product BRI2 binds APP and inhibits APP processing [3-10]. Although the familial cases caused by *APP/PSEN* mutations are classified as FAD and those caused by mutations in *BRI2/ITM2b* as Familial Danish or British dementias (FDD or FBD), recent evidence suggest that FBD and FDD share with FAD a pathogenic mechanism involving synaptic-toxic APP metabolites released during memory acquisition [11-16].

The prevailing pathogenic model for these dementias, the amyloid cascade hypothesis, posits that amyloid peptides, in forms of either amyloid plaques or oligomers, trigger dementia. In the case of AD, the amyloid peptide is Aβ, which is a part of APP and is also present in normal individuals; in the case of FDD and FBD the amyloidogenic peptides, called ADan and ABri respectively, are generated from the mutant BRI2 proteins [4,10] and are not present in normal individuals. Notably, the FDD amyloid plaques contain both Aβ and ADan*.* Based on the amyloid cascade hypothesis [17], transgenic mice carrying mutant *APPPSEN1/2* or *BRI2/ITM2b* are used to model these dementias, as over-expression is necessary to reproduce amyloidosis [18]. However, over-expression of mutant genes produce harmful effects unrelated to AD leading to erroneous information concerning pathogenesis and therapy of human diseases.

To avoid artifacts of over-expression, we generated a knock-in mouse model of FDD (FDD_KI_) that, like FDD patients [10], carries a wild type *Bri2/Itm2b* allele and the other with the Danish mutation [19]. FDD_KI_ mice develop progressive synaptic and memory deficits due to loss of Bri2, but do not develop amyloidosis [13]. BRI2 binds to APP and inhibits cleavage of APP by secretases [6-9]. Owing to the loss of BRI2, processing of APP is increased in FDD [11,12]. Remarkably, memory and synaptic deficits of FDD_KI_ mice require APP [12], and more specifically processing of APP by β-secretase during synaptic plasticity and memory acquisition [15,16]. The two products of β-processing of APP are sAPPβ and β-CTF. The latter is processed by γ-secretase to yield Aβ. Contrary to the amyloid hypothesis of AD pathogenesis, inhibition of γ-secretase did not ameliorate synaptic/memory deficits of FDD_KI_ mice [15,16]. Overall, these results provide genetic evidence that APP and BRI2 functionally interact and that APP mediates FDD neuropathology, and suggest that sAPPβ and/or β-CTF, rather than Aβ, are the toxic species causing dementia. Here, we have evaluated further the role of γ-secretase in the pathogenesis of memory deficits of FDD_KI_ mice.

## Results

### Inhibiting γ-cleavage of APP does not rescue the memory deficit of FDD_KI_ mice

**Figure 1:**
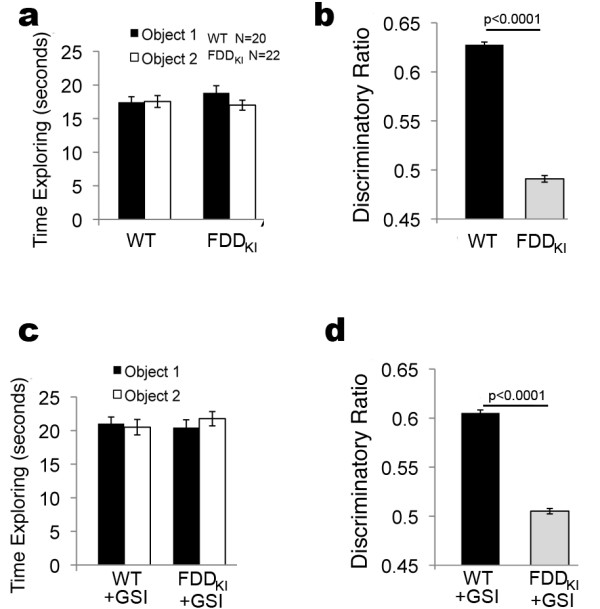
**Inhibiting γ-secretase does not rescue the memory deficit of FDD**_**KI**_**mice. a**. FDD_KI_ and WT mice were cannulated. Four weeks after cannulation mice were subjected to a NOR test. **Untreated** WT and FDD_KI_ mice spent the same amount of time exploring the two identical objects on day 1. **b**. WT mice spent more time exploring the novel object 24 hours later, showing normal object recognition (discriminatory ratio=0.63), while FDD_KI_ mice present amnesia and do not distinguish the new object from the old one (discriminatory ratio=0.5). **c**. A week after the first NOR, mice were injected in the lateral ventricle with 1μl of PBS/3μM compound-E. Injections were performed 1 hr prior to the training section, which shows that both treated groups explore equally the two identical objects. **d**. The following day, 1 hr before testing mice were injected in the lateral ventricle with 1μl of PBS/3μM compound-E. The GSI neither rescued the memory deficit of FDD_KI_ mice nor it changed memory of WT animals.

To test the role of γ-secretase in the pathogenesis of aging-dependent memory deficits developed by FDD_KI_ mice [13], a cannula was surgically implanted in the lateral ventricle of a cohort of 9-month-old FDD_KI_ mice and WT littermates. Four weeks post-surgery, we analyzed the memory deficits of FDD_KI_ mice using novel object recognition (NOR), a non-aversive memory test that relies on the mouse’s natural exploratory behavior. Prior to the NOR tests, open field studies showed, as previously reported [13], that FDD_KI_ mice have no defects in habituation, sedation, risk assessment and anxiety-like behavior in novel environments. First, the mice were studied without treatments. The NOR test showed that during training, FDD_KI_ and WT mice spent the same amount of time exploring two identical objects (Figure 1a). The following day, when one of the two old objects was replaced with a new one, WT mice preferentially explored the novel object; on the other hand FDD_KI_ mice spent the same amount of time exploring the two objects as if they were both novel to them, showing that they had no memory of the objects from the previous day (Figure 1b). This data further confirms the amnesia caused by the FDD mutation in one of the two *Bri2/Itm2b* mouse alleles.

One week later, we tested the mice again to determine whether the GSI compound-E could rescue this amnesic phenotype. To this end, these same animals were injected 1 hr before the training with 1μl of a 3μM solution of compound-E in PBS. Again, both treated WT and FDD_KI_ mice spent similar times exploring the two identical objects on day 1 (Figure 1c). One day later, mice were again injected with 1μl of a 3μM solution of compound-E in PBS 1 hr before the testing section with the new object. In agreement with what we have previously shown [15,16] the GSI neither improved memory of FDD_KI_ mice nor altered performance of WT animals (Figure 1d).

### Inhibiting γ-secretase worsens the memory deficit of FDD_KI_ mice

**Figure 2:**
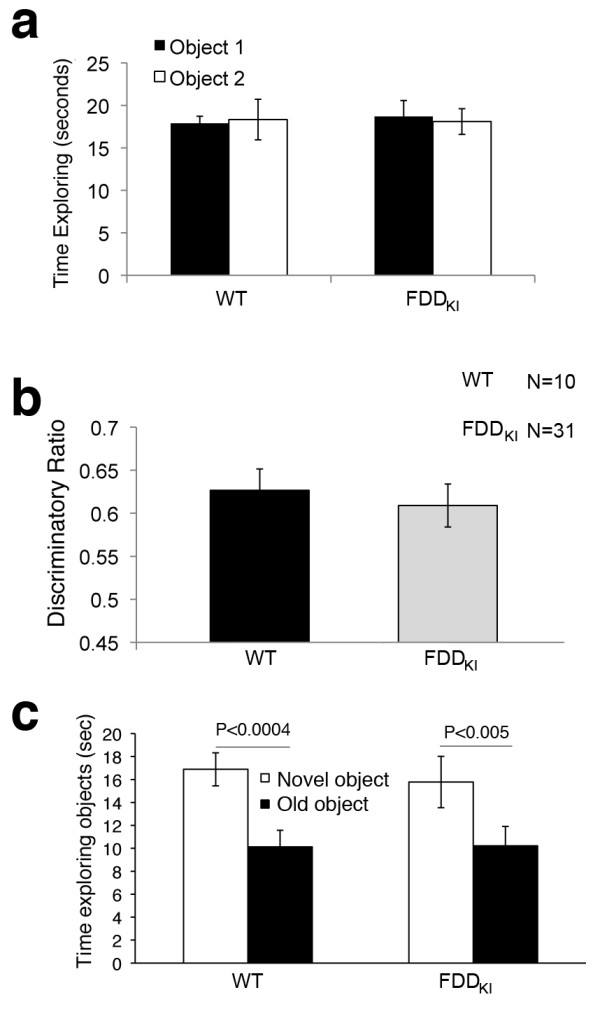
**Younger FDD**_**KI**_**mice do not present novel object recognition deficits with short retention time. a**. Also this cohort of cannulated WT and FDD_KI_ mice spent the same amount of time exploring the two identical objects in the training section. **b**. Both WT and FDD_KI_ mice spent more time exploring the novel object 4 hours later, showing that 7-month-old FDD_KI_ mice have normal object recognition when the test trial is performed with short (4 hours) retention times. **c**. Both WT and FDD_KI_ mice spent similar time exploring the objects, showing that the genotype does not affect the exploratory activity of the mice.

In the NOR paradigm used, the memory for the old objects is tested 24 hrs after the initial exposure to these objects. With this delay, FDD_KI_ mice show complete amnesia. We tested whether younger FDD_KI_ mice show memory deficits when the novel object is showed to the mice four hours after the training trial with the two identical objects. Cannulas were surgically implanted in the lateral ventricle of a new cohort of 5/6-month-old FDD_KI_ mice and WT littermates. At this age, FDD_KI_ mice already show memory deficits [13]. Three weeks after the surgery, we performed a NOR experiment with a 4 hours delay between the training test with the two identical objects, and the test trial with the new object. Again, during training FDD_KI_ and WT mice explored the two identical objects equally (Figure 2a). When these mice were tested four hours later with the new object, both 6/7-month-old FDD_KI_ and WT mice, preferentially explored the novel object (Figure 2b, c) and spend similar total times exploring each object (Figure 2c). Thus, in this experimental paradigm younger FDD_KI_ mice show no memory deficits.

**Figure 3:**
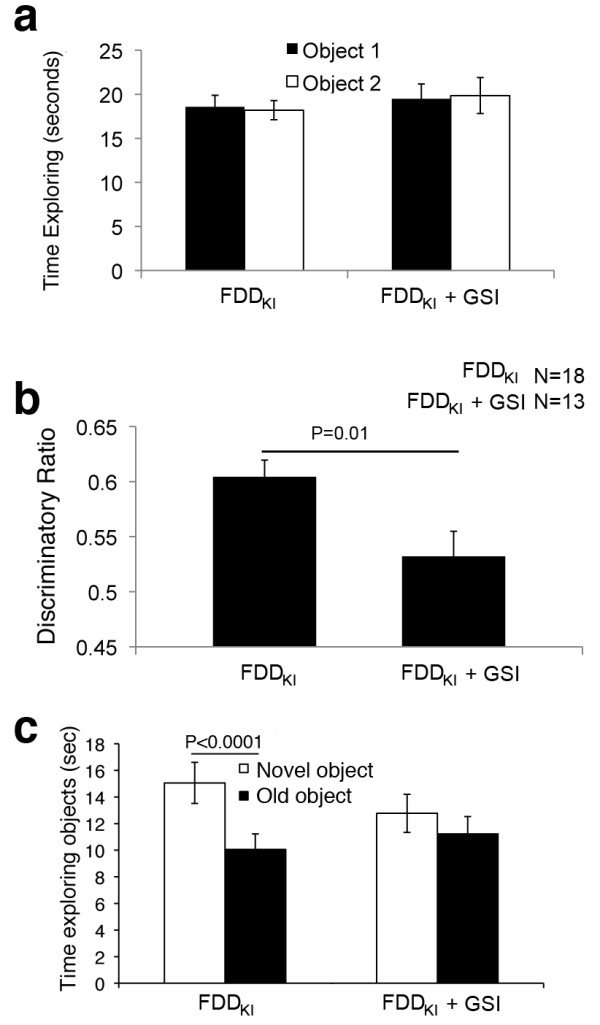
**Inhibiting γ-secretase worsens the memory deficit of FDD**_**KI**_**mice.** Mice were injected in the lateral ventricle with either 1μl of PBS or 1μl of PBS/3μM compound-E. Mice where injected once, 1 hour prior to the training. **a**. FDD_KI_ mice, regardless of whether they were injected with vehicle alone of GSI, spent the same amount of time exploring the two identical objects in the training trial. **b**. FDD_KI_ mice injected with PBS spent more time exploring the novel object 4 hours later, showing normal object recognition, while FDD_KI_ mice treated with GSI present a statistically significant novel object recognition deficit. **c**. There is no statistically significant difference in exploratory activity between treated and untreated FDD_KI_ mice.

Having established that inhibition of γ-secretase does not ameliorate memory deficits of FDD_KI_ mice (Figure 1d and [15,16]), we took advantage on this experimental setting in which FDD_KI_ mice perform equally well as WT mice, to determine whether GSI could have a detrimental effect on memory. To this end, the day after the first NOR test shown in Figure 2, FDD_KI_ mice were injected once, one hour before the training test, with either 1μl of PBS or 1μl of a 3μM solution of compound-E in PBS. GSI treated mice explored the two identical objects similarly to the PBS-treated animals (Figure 3a). When these mice were subjected to the trial test with the new object, GSI-treated FDD_KI_ mice showed a statistically significant memory deficit as compared to vehicle-treated FDD_KI_ mice (Figure 3b, c). Overall, these data show that inhibition of γ-secretase produces a worsening rather than amelioration of the memory deficit of FDD_KI_ mice. These data are consistent with the Phase III clinical trial with the GSI Semagacestat in AD patients.

### Inhibiting γ-secretase causes accumulation of APP-COOH-terminal fragments

**Figure 4:**
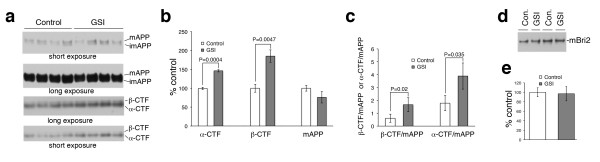
**Inhibition of γ-secretase causes an accumulation of β-CTF and α-CTF in hippocampal synaptic preparations. a.** Hippocampal synaptosomes preparations from 4 FDD_KI_ mice treated with GSI for 5 hrs and 4 FDD_KI_ vehicle-treated animals (Control) were analyzed by WB for APP and CTFs. **b**. Graph representing quantization of quadruplicate samples. The levels of β-CTF and α-CTF in vehicle-treated mice were assigned an arbitrary value of 100. The amounts of β-CTF and α-CTF in treated samples were expressed as a % of the levels in the control. Synaptic fractions from GSI treated FDD_KI_ mice express significantly more α-CTF (P=0.0004) and β-CTF (P=0.0047) than vehicle-treated samples. Similar levels of mAPP are present in both groups. **c**. The α-CTF/mAPP and β-CTF/mAPP ratios are increased in treated mice compared to vehicle-treated samples (P=0.035 and P=0.02, respectively. **d**. Synaptic fractions from both GSI treated and vehicle-treated FDD_KI_ mice express similar levels of mBri2. **e**. Quantization of mBRI2 levels in quadruplicate samples.

The dose of GSI injected (1μl of a 3μM solution) was chosen based on the following rationale. The IC_50_ for compound-E is ~240/370 pM (see manufacturer’s website). We have estimated that after injection compound-E is diluted in the CSF of the lateral ventricles ~ 200 folds (to approximately 15 nM, which is ~ 50 folds the IC_50_). Considering that clearance and distribution of the drugs in various area of the CNS further dilutes the GSI, it can be safely presumed that the concentration of the GSI in the hippocampus of mice during the course of the experiment was not excessively high. To determine whether this GSI dosage was sufficient to yield some level of inhibition of γ-secretase activity, we measured β-CTF and α-CTF, which increase when γ-secretase is inhibited. Because novel object recognition is a hippocampal-dependent memory task and synaptic activity is associated with learning and memory, we measured the levels of these APP fragments in purified hippocampal synaptosomes. As shown in Figure 4a, b, the levels of both β-CTF and α-CTF were significantly increased in mice treated for 5h with compound-E as compared to untreated animals. In contrast, the levels of mature APP (mAPP, Figure 4a, b) and mature Bri2 (mBri2, Figure 4d, e) were unaffected by the GSI. The increase in APP-CTFs in hippocampal synaptic preparations resulted in significantly higher β-CTF/mAPP and α-CTF/mAPP ratios in GSI treated mice as compared to control mice (Figure 4c). These data indicate that injection of 1μl of a 3μM solution of compound-E was sufficient to measurably inhibit γ-secretase activity and processing of β-CTF and α-CTF by γ-secretase.

## Discussion

**Figure 5:**
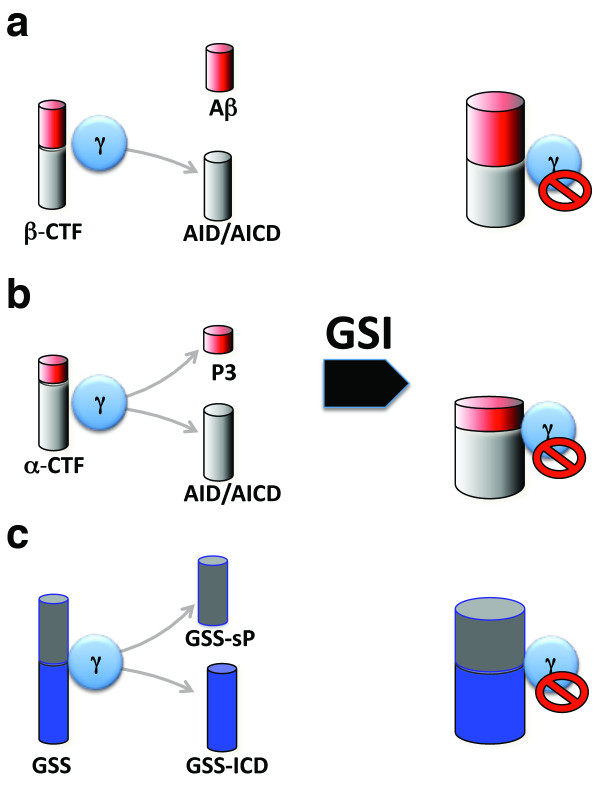
**Models explaining how GSI treatment leads to memory deficits.** GSI treatment causes accumulation of β-CTF (**a**) and α-CTF (**b**). One or both of these APP metabolites may exert a synaptic-toxic activity leading to worsening of memory. This model also suggests that the products of γ-processing of β-CTF and α-CTF (Aβ, the APP intracellular domain AID/AICD, and P3) do not play a major role in the pathogenesis of memory loss. (**c**) γ-secretase cleaves more than 40 substrates (indicated as GSS). These cleavages release an Aβ/P3 like soluble peptide (GSS-sP) and an intracellular domain peptide (GSS-ICD). The inhibition of γ-cleavage of these other substrates will lead to a reduction in GSS-sP and GSS-ICD and an accumulation of GSS. These changes for one or more of these other GSS may participate in or cause the worsening of memory loss in FDD_KI_ mice.

We have previously shown that the synaptic plasticity and memory deficits in FDD are mediated through production of sAPPβ and/or β-CTF during LTP and memory acquisition. The failure of GSI to rescue the deficits of 9/10-month-old FDD_KI_ mice ([15,16] and Figure 1d) suggests that Aβ, P3 and AID/AICD, the metabolites derived from γ-cleavage of APP (Figure 5a, b), are not involved in these pathogenic processes. Younger FDD_KI_ mice showed no memory deficits when subjected to NOR tests with a shorter (4 hours) retention time. Interestingly, GSI treatment of these mice provoked a memory deficit, which correlates with an accumulation of β-CTF and α-CTF. Altogether, the data indicate that reducing γ-secretase activity is detrimental rather than beneficial in our mouse model of dementia. The evidence that *PSEN1* and *PSEN2* FAD mutations cause loss of γ-secretase function and that loss of Presenilins’ function cause synaptic plasticity deficits, memory defects and neurodegeneration in mice [20-23] is consistent with these results.

The accumulation of β-CTF and α-CTF caused by GSI treatment may prompt worsening of memory in FDD_KI_ mice (Figure 5a, b). However, γ-secretase cleaves more than 40 substrates. Therefore, the toxic effect caused by GSI treatment may arise from inhibition of processing of other γ-secretase substrates (Figure 5c). These two hypotheses do not need to be mutually exclusive. Our data are concordant with two other set of evidence. First, a phase III clinical trial with Semagacestat, a γ-secretase inhibitor, was halted because Semagacestat rather than slowing disease progression caused a worsening of clinical measures of cognition and the ability to perform activities of daily living. Second, prolonged (8 days) treatment with GSIs produced no positive effects on memory deficits of older APP transgenic mice, and induced cognition deficits in both young APP transgenic mice and mice. These effects also correlated with accumulation of α/β-CTFs [24].

In conclusion, this study suggests that targeting Aβ production may be ineffective or, perhaps, detrimental. Importantly, our results once more show that our FDD_KI_ model is useful to study pathogenic mechanisms of dementia and to test in preclinical studies the efficacy of candidate disease modifying drugs for AD.

## Material and methods

### Mice

Mice were generated and maintained at the Animal facility of the Albert Einstein College of Medicine. Mice were handled according to the Ethical Guidelines for Treatment of Laboratory Animals of Albert Einstein College of Medicine. The procedures were described and approved in animal protocol number 200404.

### Reagents

Compound-e was purchased from (Calbiochem).

### Brain cannulation and injections

Dr. Xiaosong Li at the Animal Physiology core of the Albert Einstein College of Medicine surgically implanted the cannula. Compound-E or PBS was delivered at the rate of 1 ml per minute using a CMA 400 syringe pump.

### Open field and novel object recognition

The mice were acclimated to the testing room for 30 min after being moved. Each mouse was placed into a 40 cm X 40 cm open field chamber with opaque walls, 2ft high. Each mouse was allowed to habituate to the normal open field box for 10 min, and repeated again 24 h later, in which the video tracking system (HVS 2020; HVS Image) quantified various locomotor parameters: total distance travelled, number of entries into, distance travelled in, and time spent in the centre of the locomotor arena. As previously reported [13], open field studies showed that FDD_KI_ mice have no defects in habituation, sedation, risk assessment and anxiety-like behavior in novel environments.

Novel object recognition began 24 h after the second open field session, and was performed as previously described [13,25]. Briefly, NOR consisted of two sessions, either 24 h (Figure 1) or 4 h (Figures 2 and 3) apart. In the first session, the mice were placed into the open field chamber with two identical, non-toxic objects, 12 cm from the back and sidewalls of the open field box, and 16 cm apart from each other. A 8 min session, in which the time exploring each object was recorded; an area 2 cm2 surrounding the object is defined such that nose entries within 2 cm of the object were recorded as time exploring the object. The animal was then returned to its home cage, and either 24 or 4 h later, placed into the open field box again. This time, there were two new objects, one identical to the previous objects, and one novel object. The mice were given another 6 min to explore, and the amount of time exploring each object was recorded. Mice that spent <7 s exploring the objects were omitted from the analysis [25]. Results were recorded as an object discrimination ratio (ODR), which is calculated by dividing the time the mice spent exploring a novel object, divided by the total amount of time exploring the two objects.

### Synaptosomes preparations and Western blot analysis

For synaptic preparations, isolated hippocampi were homogenized (w/v= 10 mg tissue/100ml buffer) in Hepes-sucrose buffer (20 mM Hepes/NaOH pH 7.4, 1 mM EDTA, 1 mM EGTA, 0.25 M sucrose) supplemented with protease and phosphatase inhibitors. Homogenates were centrifuged at 800 g for 10 min. The supernatant (S1) was separated into supernatant (S2) and pellet (P2) by spinning at 9,200 g for 15 min. P2 contains the crude synaptosomal fraction. Synaptosomes fractions were analyzed by western blot using the following antibodies: α-APP (22C11/Chemicon) to detect mAPP and imAPP; α-APPCTF (Invitrogen/Zymed) to detect α–CTF and β-CTF; α-BRI2 (Santa Cruz) to detect mBri2.

### Image scanning and analysis

WB images were scanned with Epson perfection 3200 Photo scanner and were analyzed with NIH ImageJ software.

### Statistical analysis

All data are shown as mean s.e.m. Statistical tests included two-way ANOVA for repeated measures and *t*-test when appropriate.

## Misc

Robert Tamayev and Luciano D’Adamio contributed equally to this work

## Authors’ contributions

LD generated the mice. RT performed behavioral experiments. LD performed hippocampal preparations and western blot analysis. LD designed research and wrote the paper. Both authors read and approved the final manuscript.
